# The Case Against Betadine Irrigation of Breast Implant Pockets

**DOI:** 10.1007/s00266-022-03123-y

**Published:** 2022-10-04

**Authors:** Eric Swanson

**Affiliations:** Swanson Center, 11413 Ash St, Leawood, KS 66211 USA

*Level of Evidence V* This journal requires that authors assign a level of evidence to each article. For a full description of these Evidence-Based Medicine ratings, please refer to the Table of Contents or the online Instructions to Authors www.springer.com/00266.

Venkataram et al. [1] promote Betadine (Avrio Health, Stamford, CT) irrigation of breast implant pockets as a proven method to reduce the risk of capsular contracture. The authors call for universal adoption of Betadine-containing antimicrobial breast pocket irrigation to reduce capsular contracture risk and “other device-associated infection.” The discussant, and frequent collaborator with the senior author, believes that the evidence is conclusive [2]. This recommendation stands in stark contrast to (unreferenced) recent recommendations that this practice should be abandoned [3–6]. Antimicrobial irrigation is a highly relevant topic for any plastic surgeon who inserts breast implants and one that merits a scientific analysis. Is this erstwhile practice worthy of its continuation, or is it time to reconsider?

Importantly, the authors do not mention (or deny) the fact that Betadine 10% is nonsterile [7, 8]. This antimicrobial solution is not recommended for internal use. Bottles of Betadine are labeled “Topical Bactericide.” The warnings, “Antiseptic Nonsterile Solution” and “For External Use Only,” appear on the bottles [7]. Despite these warnings, Adams dismisses nonsterility as “urban legend” [9]. Adams and Calobrace [10] believe that the inside of the bottle is sterile, if not the outside. However, this statement is not true for the “stock” [11] 10% povidone-iodine solution [6].

There are other important deficiencies (Table 1). The authors write, “in August 2017 the FDA [U.S. Food and Drug Administration] finally lifted the restriction of betadine for use for Allergan implants based on long-term data supplied the directions for use (DFU) for Betadine (*sic*), making it once again an on-label acceptable use in breast implant pocket preparation/irrigation. What was formerly an off-label practice can now be practiced openly for bacteria/biofilm mitigation and antimicrobial prophylaxis” [1].

**Table 1:** Factual deficiencies in 2022 article promoting betadine irrigation^1^

No.	Deficiency
1. 2. 3. 4. 5. 6. 7. 8. 9. 10. 11. 12. 13. 14. 15. 16. 17. 18.	No recognition that Betadine is nonsterile and unapproved for use in breast wounds. Erroneous statement that the FDA has approved Betadine for use in breast implant pocket irrigation. Unsubstantiated claim that BIA-ALCL is caused by a Gram-negative microbiome. “Prospectively recorded data” suggests a prospective study. This was a retrospective study. Authors mention a controlled setting. The study was not controlled. The title indicates 20-year follow-up. This represents the maximum follow-up time. The mean was 6 years. The title mentions “enhancing patient outcomes.” There were no patient-reported outcome data. The criteria for diagnosing a capsular contracture were not reported. Incorrect claim that the study was exempt from IRB oversight. There was no IRB approval or waiver. An unreported number of patients did not receive Betadine irrigation but were included in the evaluation. The number of patients who returned in follow-up for evaluation and the follow-up times are not provided. Advocates of the 14 points do not follow the points consistently. The lack of evidence supporting the 14 points is not discussed. The authors do not reference any studies that do not support Betadine irrigation, including recent reviews. Referenced studies include antibiotic irrigation of other body cavities that are dissimilar. The authors do not discuss the risk of microbial contamination of this nonsterile irrigation solution. The cytotoxicity of Betadine is dismissed. Financial conflicts are not disclosed.

*BIA-ALCL*, breast implant-associated anaplastic large cell lymphoma; *FDA*, US food and drug administration; *IRB*, institutional review board.

This statement is misleading. First, the directions for use for Betadine include no mention of breast implant pocket irrigation and never have. On the contrary, this antimicrobial solution is intended “for external use only” [7]. Second, Betadine 10% solution is not approved and has never been approved by the FDA for anything [6]. The statement “pocket irrigant used with signed off-label consent” [1] is therefore confusing. The authors’ reference is to an August 28, 2017, communication from the FDA approving a labeling change for Natrelle breast implants (Abbvie Inc., North Chicago, Ill.), not Betadine. The FDA statement reads, “Approval for changes to the labeling including 1) the removal of the Betadine warning against breast implant exposure to Betadine brand povidone-iodine 10% (applicable to generic versions as well) from the patient and physician labeling, and 2) modifications to the language in the physician and patient labeling regarding the potential risk of breast implant-associated anaplastic large cell lymphoma (BIA-ALCL)” [12]. This labeling change acknowledged that Betadine irrigation was unlikely to affect the implant shell integrity [13]. It was not an endorsement by the FDA that “what was formerly an off-label practice can now be practiced openly for bacteria/biofilm mitigation and antimicrobial prophylaxis” [1, 13].

Remarkably, the authors report, “we prefer the use of BT [Betadine] over NB-TAB [non- Betadine Triple Antibiotic] as it has stronger gram-negative coverage, in the light of recent revelations that BIA-ALCL is caused by chronic inflammation by a Gram-negative microbiome” [1]. No reference to such recent revelations is provided. On the contrary, a recent publication summarizing the findings of the 2021 Consensus Conference on BIA-ALCL reported no evidence of a microbiological etiology for BIA-ALCL [14]. At one time, *Ralstonia pickettii* was thought to be the culprit [15]. However, this association has not been reproduced [16] and any possible link is now regarded as either fictitious or the result of laboratory contamination [14, 16]. The microbiome of a capsule affected by BIA-ALCL is not microbiologically distinct from the normal capsule [17]. Chronic inflammation and particles are now thought to be implicated, not infection [14].

In scientific writing, proper language is important so as not to create ambiguity. The authors state that their data were collected prospectively, which is the way data are always recorded [18]. This was actually a retrospective study [1]. A retrospective study is undertaken once the data have been collected [18]. The difference is important. Prospective studies are preferred for several reasons, including the fact that the outcome is unknown (the data are yet to be collected) when the study is undertaken, so that researchers are less likely to be biased [18].

The authors report a “controlled single surgeon setting” [1]. In this study, all patients received antimicrobial solution; there was no control group. Stating that the setting was “controlled” might cause confusion. The title mentions a 20-year follow-up. One might think that these patients were followed for 20 years. The study period concluded in 2017. Why not evaluate another 5 years of data? No manufacturer information is provided. The implant type (saline versus silicone gel) is not reported.

The title includes the words, “enhancing patient outcomes” [1]. However, no outcome data are reported apart from capsular contracture rate. Outcome studies typically report patient-reported outcomes, obtained from surveys [18]. One must be cautious when using the word “proven” in the title and repeated 10 times in the article, especially when discussing capsular contracture, which has an unknown etiology.

The authors claim that their study was exempt from Institutional Review Board approval under Title 45, Part 46.104(d)(4) of the Code of Federal Regulations [19]. Exemptions apply to: educational techniques, observations of public behavior, behavioral interventions, secondary research of existing data or specimens, demonstrations projects, and taste research [20]. It is not clear that this study fits one of these exemptions. The authors state that the study was conducted following the guiding principles of the Declaration of Helsinki [1]. The principles of the Declaration of Helsinki most relevant to plastic surgery include the need for proper informed consent prior to enrollment, the need for approval by an independent ethical review board, the need to disclose negative as well as positive results, and the need for proper disclosure of all sources of funding, institutional affiliations, and conflicts of interest [21]. Review and approval by an institutional review board is required for all projects that involve research with human subjects [20]. Additionally, the approval must precede the start of the research [20]. Lack of institutional review board approval violates federal regulations and can result in a report of noncompliance to university officials, if applicable, as well as the Office of Human Research Protections in Washington DC [22].

The “Betadine triple preparation” is described as 50 mL Betadine, 1 g of basil, 80 mg gentamicin, and 500 mL normal saline [1]. The authors no doubt meant to write 1 g of cefazolin, not the herb [23]. The irrigation solution used was either diluted Betadine (50% betadine or “betadine triple”) or a non-betadine triple antibiotic regimen [1]. The numbers of patients treated with each solution are not reported. Patients are not subdivided into groups treated with or without Betadine [1]. This deficiency makes it impossible to accurately assess the capsular contracture rate in patients treated with Betadine irrigation, simply because an unknown number of patients were not treated with Betadine irrigation.

The authors state that their practice is to contact patients virtually each year. Outgoing emails do not count as follow-up. In fact, no information is provided regarding how many patients were actually seen in follow-up and at what times. The inclusion rate is missing. The method for diagnosing a capsular contracture is not described. The mean follow-up, 6 years, applies to the subset of patients that came in for a visit [1]. The number of capsular contractures is compared to the total number of patients who had breast augmentations, the majority of which are lost to follow-up [24]. This comparison contributes to an exceptionally low reported capsular contracture rate (< 1%).

Cosmetic breast patients are notorious for not keeping long-term follow-up appointments [24]. In fact, this is a problem for all manufacturer core studies that try to maximize follow-up. Core studies, which are regarded as most robust, report capsular contracture rates in the range of 8- 19%, [25] with no recent trend [26].

The authors state that the “14 point plan steps were followed in all cases,” referencing the original 2013 publication detailing the 14 points [27]. However, it is not clear that these points were always observed. Publications by Adams in 2006 and 2008 include no mention of nipple shields (point 3) [23, 28]. A recent video does not show the senior author switching out instruments and drapes prior to handling the implant (point 9) [29]. The authors state that they have never used an introduction sleeve (point 8) [1]. Adams uses drains (violating point 13) when inserting new implants at the same time that old capsules are removed [29]. Adams explains, “these principles you can follow but they are not rote principles, so you do whatever you need to do” [29]. Here, the authors emphasize, “It should be noted that doing these the same way, every time, for every case is the key to this process-oriented approach” [1]. The 14 points have evidently changed. There is no longer a requirement for an introduction sleeve [1]. There is no provision to replace the instruments and drapes during the case; “clean” instruments will suffice [1].

The authors write, “A similarly low rate of BIA-ALCL was observed in this study like other publications using the same technique,” referencing the 2017 publication by Adams et al. [30]. This publication was actually a survey, as opposed to a clinical study, although that important fact was not disclosed, among other problems [31]. Not surprisingly, the authors of that publication did not all use the same technique; they frequently deviated from the 14-point plan [32]. The reported 11.7-year mean follow-up time was skewed by one Swedish co-author who claimed a 14-year mean follow-up time among 16,000 patients, which is highly questionable [31]. Like the present study [1], there was no information regarding how many patients returned for follow-up visits and when [31, 32]. The authors simply assumed that there were no BIA-ALCL cases because they were unaware of any [31].

The authors acknowledge that the vital question is whether or not to irrigate the pocket with antimicrobial solution [1]. There is no evidence that any of the other points have any relevance to capsular contracture or BIA-ALCL. It has always been a curious deficiency of the 14 points that “avoidance of textured implants” is not included—the one point that makes a difference [31, 32]. The authors recognize that textured implants carry more bacteria, which they seek to minimize using antimicrobial irrigation [1]. Moreover, textured implants are now considered causative in the etiology of BIA-ALCL [14].

For years, industry has supported an infectious etiology for BIA-ALCL as opposed to a device-related etiology, including support for the 14 points [26, 31]. Financial disclosures by conflicted authors are essential, but are frequently unreported [1, 9, 11, 26, 31, 33]. According to the Open Access payments site, Adams has received over $112,000 from Allergan during the period 2016 to 2019 [34].

Venkataram et al. [1] consider the matter of antimicrobial irrigation settled. They reference 21 other supportive studies, although one of these studies, a review article, cited limited evidence of poor quality [35]. They reference 9 additional studies of antimicrobial irrigation in unrelated applications (e.g., a 1992 Russian-language publication with an English translation: “the affinity lavage of the abdominal cavity in diffuse peritonitis with liquid sorbents based on cross-linked dextrans”) [36]. Of course, evidence-based medicine is concerned about the quality of publications, not just the number [18]. The authors do not reference publications that oppose their conclusion [3–6, 16, 17, 25, 26, 31, 32, 37, 38]. The series by Blount et al. [39] is often cited as evidence of a 10-fold reduction in risk, [1, 10] without recognizing that a significant risk reduction disappeared on multivariate analysis [39]. The authors do not reference 2 recent systematic reviews, including 1 meta-analysis, that found that antimicrobial irrigation is unhelpful [37, 38]. A systematic review using the Methodological Index for Non-randomized Studies (MINORS) and Cochrane risk-of-bias tool revealed poor methodologic quality and selection bias among a heterogeneous patient population, predisposing to an erroneous association between antibiotic irrigation and capsular contracture [38]. Scientifically, one can accept contradictory evidence or reject it, but not ignore it. Indeed, even if there were a benefit, surgeons cannot, ethically or legally, use nonsterile fluids in surgery to irrigate open noninfected wounds [6].

Any nonsterile solution introduced into an open wound creates a risk of contamination [3, 6, 40, 41]. In the 1970s, it was assumed that antiseptics were free of microbial contamination because of their pharmacologic activity [41]. In 2013, the FDA addressed safety issues after receiving reports of infections that were confirmed to have been caused by contaminated topical antiseptics, including povidone-iodine [40, 41]. These infections are likely under-reported [41].

In 1985, Lineaweaver et al. [42] evaluated the effect of 1% povidone-iodine when applied to cultured human fibroblasts, finding no fibroblast survival at 24 hours (i.e., 100% cytotoxicity). The same result was obtained when solutions of 0.25% acetic acid, 0.5% sodium hypochlorite, and 3% hydrogen peroxide were used. The authors concluded that these solutions are unsuitable for use in wound care. Unfortunately, this research was evidently not considered by Burkhardt et al., when these plastic surgeons introduced this cytotoxic solution to breast surgery in 1986 [43]. Breast pockets differ from other types of wounds. The environment is nonsterile [3]. Numerous harmless and possibly protective commensal organisms reside in the breast [3]. A “shotgun” approach is likely to eliminate resident organisms, altering the microflora in ways that are unlikely to be beneficial [3]. If the surgeon adheres to ordinary sterile technique, the microbiome is unchanged, and there is no opportunity for nonresident pathogens to be introduced [6].

Irrigation of breast pockets with nonsterile Betadine solution has gone on for too long. This unauthorized practice, [7, 8, 40] of questionable efficacy, [3, 6, 35, 37, 38] and proven cytotoxicity, [42] should be abandoned [6]. Longstanding practices sometimes need to change [26]. First, do no harm.
